# The Acute Effect of Magnesium Supplementation on Endothelial Function: A Randomized Cross-Over Pilot Study

**DOI:** 10.3390/ijerph18105303

**Published:** 2021-05-17

**Authors:** Caitríona Murphy, Jennifer Byrne, Jennifer B. Keogh, Michelle L. Headland, Peter M. Clifton

**Affiliations:** 1 Clinical and Health Sciences, University of South Australia, Adelaide, SA 5001, Australia; caitriona49@hotmail.com (C.M.); jenniferbyrne12@hotmail.com (J.B.); jennifer.keogh@unisa.edu.au (J.B.K.); michelle.headland@unisa.edu.au (M.L.H.); 2 Department of Biological Sciences, Technological University Dublin, D07 EWV4 Dublin, Ireland

**Keywords:** magnesium 1, flow mediated dilatation 2, endothelial function

## Abstract

Magnesium (Mg) deficiency might be a catalyst in the process of endothelial dysfunction, an early event in the pathogenesis of atherosclerosis. The aim of this study was to determine the acute effect of an oral Mg supplement as compared to control on endothelial function assessed by flow-mediated dilatation (FMD). Nineteen participants (39 years, body mass index (BMI) 22.9 kg/m^2^) completed this randomized cross-over study. Blood pressure (BP) and FMD were measured and blood samples were taken before participants drank 200 mL water, with or without an over the counter Mg supplement (450 mg and 300 mg for men and women). Measurements were repeated at 60 and 120 min. There was a statistically significant two-way interaction between treatment and time on serum Mg (*p* = 0.037). A difference of −0.085 mm in FMD was observed 60-min post drink in the control group, as compared to baseline FMD, and no difference was observed in the supplement group as compared to baseline. Despite the non-significant interaction between treatment and time on FMD, once adjusted for baseline, the difference seen in the control group and the lack of change in the supplement group at 60 min post-drink suggests that Mg might attenuate the reduction in FMD post-prandially.

## 1. Introduction

Cardiovascular disease (CVD) is the leading cause of death worldwide with 31% of all global deaths attributed to CVD [1]. Magnesium (Mg) is emerging as a nutrient of importance in cardiovascular health [2,3,4,5,6]. Low serum Mg is associated with increased coronary artery disease (CAD) risk [6]. In a large study of 14,446 participants over 27 years, during which 2131 cases of CAD cases were seen, it was observed that low serum Mg was associated with higher CAD [6]. Dietary magnesium might reduce all-cause mortality as seen in a recent meta-analysis with each additional intake of 100 mg/d of dietary magnesium being associated with a 6% risk of all-cause mortality. [7]. It is unclear what the potential mechanism for this effect might be, but there is evidence that fruit and vegetable intake is inversely associated with CVD risk [8]. Several minerals might contribute to this beneficial effect including reducing sodium [9,10,11,12] and increasing potassium intakes [13,14,15], and increased Mg intake [3].

The main underlying cause of CVD is atherosclerosis with endothelial dysfunction as an early event in its pathogenesis [16,17]. Flow-mediated dilatation (FMD), an ultrasound measurement of the brachial artery, is a non-invasive technique for measurement of endothelial function and is a useful surrogate measure of the endothelial function in coronary arteries [18,19]. Higher dietary Mg intake and serum Mg concentrations are inversely associated with endothelial dysfunction and markers of inflammation [20,21,22]. A recent meta-analysis suggests that chronic Mg supplementation over 6 months might improve FMD in overweight or older individuals [23,24]. However, this was not confirmed in a randomized placebo-controlled 24-week study with 350 mg of Mg in 52 overweight and obese subjects. FMD was measured in the fasting state and whether there was any effect on post-prandial FMD, remains unknown. Therefore the aim of this study was to investigate the acute effect of Mg supplementation on post-prandial FMD to determine its effect on endothelial function in the post-prandial state. 

## 2. Materials and Methods

### 2.1. Volunteers

Participants aged 18–75 years, BMI 18–35 kg/m^2^ and SBP < 140 mm, DBP < 90 mm, were recruited via public advertisement, using flyers and social media posts. They were screened for eligibility prior to attending their first visit. Participants were weight stable in the preceding 6 months, and were not currently taking cholesterol or antihypertensive medication, non-steroidal anti-inflammatory medications, drugs affecting endothelial function, or folate supplementation. 

### 2.2. Trial Protocol

At a screening visit in which participants eligibility for the trial was confirmed, body height and weight and blood pressure (BP) was measured, and instructions on how to complete a 3-day food diary and 24-h urine collection were given. In two subsequent visits, measurements of BP and flow-mediated dilatation (FMD) were taken, and a blood sample was collected at time-point 0 before consuming a 200-mL drink, with or without an Mg supplement in a randomized manner. These measurements were collected again at 60- and 120-min after drink consumption. Supplementation was over-the-counter Mg citrate supplements ([25] Wellness, Australia) either 3 × 150 mg for men or 2 × 150 mg for women. The dose was equivalent to 300 mg/day for women and 450 mg/day for men, with the aim of meeting the Australian recommended daily intake (RDI) of 400–420 mg/day and 310–320 mg/day for men and women, respectively [26]. Given that the RDI might be too low, given the rising body weights, we calculated an Mg supplement dose /kg variable and used this as a covariate in the analysis [27]. This trial was approved by the University of South Australia’s Human Research Ethics Committee (approval number: 0000035834) and is registered with the Australian New Zealand Clinical Trials Registry (ANZCTR; number 12617000160336). All participants gave written informed consent. An honorarium of $150 was offered to participants once they had completed all study visits. 

An online generated random number allocation sequence was used to randomize the order of the visits (www.randomization.com; accessed on 30 January 2017). Randomization was performed by a research team member that did not complete the FMD measurements and the code was stored on a password-protected computer. 

At the screening visit, participants’ body height was measured to the nearest 0.01 cm (SECA, Hamburg, Germany), whilst barefoot. Body weight was also measured via electronic scales to the nearest 0.05 kg (SECA Hamburg, Germany) at each study visit. Participants wore light clothing and wore no footwear.

Participants were asked to complete a 3-day weighed food diary (one weekend day and two weekdays) prior to their first visit, to determine their habitual dietary intakes of Mg. Analysis of the food diaries was conducted via the Foodworks Profession Edition 2007 (version 5; Foodworks Professional Edition; Xyris Software, Highgate Hill, Australia). They also collected a 24-h urine sample, prior to their first visit. A total weight of the 24-h urine sample was recorded, with a 20–30 mL aliquot sent to an accredited laboratory (SA Pathology, Adelaide, South Australia) for analysis of urinary Mg and creatinine concentration. 

Blood pressure was measured using an automated sphygmomanometer (SureSigns V3; Philips, North Ryde, Australia) with participants seated. A series of 4 consistent measures were obtained, within range of 10 mm, with the first reading discarded. The average of the remaining measurements was calculated. Measurements were taken at time point 0, and then at 60 min and 120 min, following the drink at visit 1 and 2. 

### 2.3. Flow Mediated Dilatation (FMD)

Flow-mediated dilatation (FMD) measurements were performed by a single trained operator (PMC), in a quiet temperature-controlled room. All participants were fasting. Endothelium-dependent FMD of the right brachial artery was measured in the longitudinal plane, above the antecubital fossa, with an 8.8-MHz linear array transducer (GE Logiq 5). The brachial artery diameter was measured before and after forearm ischemia caused by inflation of a sphygmomanometer cuff applied to the right forearm, 2-cm below the olecranon process to 200 mmHg, for 5 min. The operator was blinded to participant condition and the measures were recorded at time-point 0, as well as 60- and 120-min post drink ± supplementation consumption. All recorded images were stored offline for analysis at a later point, and encoded to ensure blind analysis. 

Ultrasound images were recorded at a rate of 30 frames per second, with screen capture software (Debut Video Capture Software Professional V1.82; NCH Software, Canberra, Australia), and were analyzed with edge-detection software (Brachial Analyser for Research V6.1.3; Medical Imaging application LLC, Coralville, Iowa, USA), to determine artery diameter (mm) values for both baseline and deflation. Baseline was defined as the average 15-s pre inflation, and peak diameter was determined as the maximum diameter post-cuff release. The absolute change in artery diameter (mm) was then calculated as the difference between the two. 

### 2.4. Serum Magnesium

Blood samples were collected via cannular insertion into the left brachial vein at each study visit. Samples were collected at baseline, and then 60 and 120 min following the drink ± supplement for analysis of Mg. Two × 8 mL serum separator clot activator tubes were collected and left to clot for 30 min at room temperature. One tube was then centrifuged at 4000 RPM for 10 min (Universal 32R; Hettich Zentrifugen, Tuttlingen, Germany) and aliquots of the serum were stored at −80 degrees Celsius, with the other delivered to SA Pathology, Adelaide, South Australia for analysis.

Based on a previous studies 32–35 subjects would be required to detect a mean difference in FMD of 0.04 mm (α = 0.05; 80% power) between treatments. A pilot study was conducted (4 males, 17 females) to determine the power needed for a larger study and to explore the protocol to determine its efficacy and if a larger study was needed.

### 2.5. Statistics

Data were analyzed using the IBM SPSS software (version 21; IBM, Chicago, IL, USA). Significance was set at *p* < 0.05. A two-way ANOVA with repeated measures (with treatment and time as the within-subject factors) was used, with and without covariates including SBP, DBP, MAP, age, BMI, and serum Mg, Mg dose/body weight in Kg, and gender as a between-subjects’ factor. Spearman Rho correlation analyses was used to assess the association between dietary Mg intake and urinary Mg excretion, and the association between dietary Mg intake and baseline FMD. A backwards linear regression was used to explore the association between FMD, age, and dietary variables, including saturated fat, polyunsaturated fat, and dietary Mg intake and supplement dose in mg/kg body weight. All data are expressed as mean ± SD or median ± inter-quartile (IQR) as appropriate.

## 3. Results

Twenty-one healthy volunteers were enrolled in the study, and 19 completed it. Baseline characteristics are outlined in Table 1. FMD, SBP, DBP, and MAP were analyzed for all completers, however, several serum MG values were missing due to blood samples not being successfully collected. Weight was not different between visits. 

### 3.1. Dietary Intake

Table 2 outlines the usual dietary intake levels of Mg, saturated fat, and PUFA, as recorded by 3-day weighed food records, 53% of participants did not meet the RDI for Mg for their respective age and gender, and 26% did not meet the estimated average requirement (EAR) for Mg for their respective age and gender.

### 3.2. Flow-Mediated Dilatation

**Figure 1:**
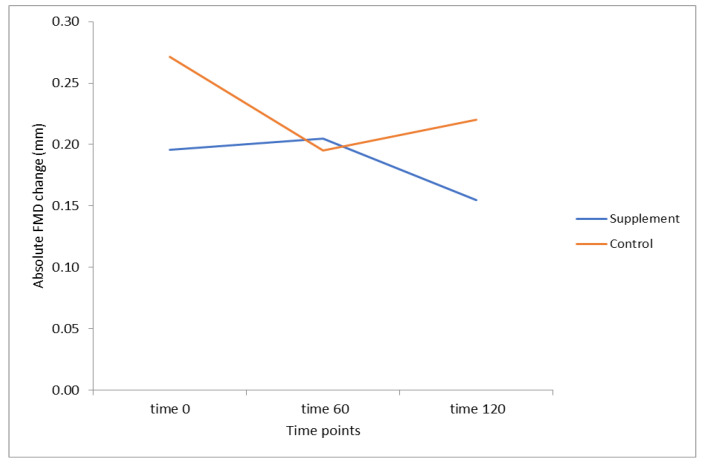
The effect of Treatment * Time Interaction on Absolute FMD.

There was no interaction between treatment and time (60 and 120 min) for absolute FMD (*F* (2, 36) = 1.371, *p* = 0.27). The main effect of treatment showed a difference in absolute FMD between treatments (*F* (2, 18) = 4.948, *p* = 0.04), which was due to average time-point 0 (pre-supplement) differences in absolute FMD of 0.076 mm, between the supplement and the control group (*p* = 0.02) (Figure 1). Time by treatment for all 3 time-points was not significant when the difference between baseline FMD was added as a covariate. Similarly, there was no treatment by time effect when examining baseline and 120 min There was no significant effect of age, gender, BMI, or serum Mg (between groups or time-points) or Mg dose in relation to body weight, when used as covariates. At time-point 60, a difference of −0.085 mm was observed in the control group as compared to time-point 0, with no change observed in the supplement group across the same time-frame.

### 3.3. Blood Pressure and Mean Arterial Pressure

There was no interaction between treatment and time on SBP or DBP. No changes were observed between treatments or any time-point for either SBP or DBP. Age, gender, or differences in serum Mg (between groups or time-points) had no impact on SBP or DBP. No interaction between treatment and time was observed for MAP, and furthermore there were no differences in MAP between treatments or time-points *F* (1, 18) = 0.509, *p* = 0.49, *F* (2, 36) = 0.82, *p* = 0.45, respectively. Age, gender, or differences in serum Mg (between groups or time-points) had no effect on MAP.

### 3.4. Serum Magnesium

There were no differences in serum Mg concentrations at time 0 or 60 min at either of the two visits, but there was significant difference at 120 min during the intervention visit (mean = 0.090 mmol) as compared to the control visit (mean = 0.871 mmol), *p* = 0.003. At the intervention visit, serum Mg increased from 0.86 ± 0.06 mmol pre-intervention, to 0.91 ± 0.06 mmol 120 min post-supplementation (*p* < 0.001). 

### 3.5. Correlations

As there was no significant difference between absolute FMD in the supplement vs. control days, an average time-point 0 absolute FMD was calculated and used in the correlational analysis. 

**Figure 2:**
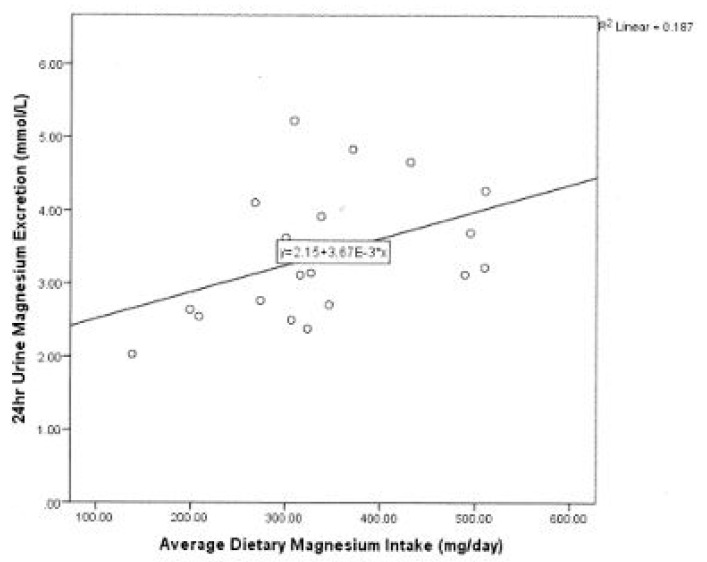
Association between Urinary and Dietary Magnesium.

Initial analysis showed a moderate positive correlation between average dietary Mg intake and 24-h urinary excretion in the cohort (r_s_ = 0.5, *p* = 0.029) (Figure 2) There was no significant correlation between average dietary Mg intake and time-point 0 absolute FMD (*p* = 0.435). Expressing Mg intake as a function of body weight did not change the results.

### 3.6. Linear Regression Analysis

Backwards linear regressions were run to predict baseline absolute FMD from age, average dietary Mg, saturated fat, and PUFA intake. Age, average dietary Mg, saturated fat, and PUFA did not predict baseline FMD. The addition of age as an independent variable in the model explained 25.8% of the variability of time-point 0 absolute FMD, the equivalent to an increase in age of 1 year being associated with a decreased in time-point 0 absolute FMD of 0.004 mm/year.

## 4. Discussion

Our findings suggest that FMD was not different at one or two hours after a single dose of Mg in water, as compared to water alone in healthy adults. It is of interest that Mg appears to attenuate the post-prandial reduction in FMD observed after water, and warrants speculation that Mg supplementation potentially attenuates the decrease in endothelial function seen in the post-prandial state. When serum Mg was accounted for as a covariate, the effect of the interaction between intervention and time on absolute FMD approached significance, further suggesting that there is a potential acute effect of Mg supplementation on FMD over time, and that perhaps a larger sample size would clarify this. Sample size calculations showed that 79 participants would be needed in a cross-over study design to see a significant difference between an Mg supplement (300 mg for women or 450 mg for men) and no supplement, as used in the present study. The results of this pilot study suggest a potential role for Mg in the attenuation of post-prandial endothelial dysfunction, warranting the need for further research in this area.

### 4.1. Dietary Influences on FMD

Water alone was shown to impair FMD by 15% in healthy participants [28]. Other minerals and nutrients were also shown to have an effect on FMD. High salt intakes have acute adverse effects on vascular dilatation in the postprandial state [10], and the addition of potassium to a high-sodium meal attenuates the sodium-induced, post-meal reduction in endothelial function, as assessed by FMD [14]. A decrease in FMD was observed 1 h after a 75 g glucose drink, which was restored within 4 h [29,30,31]. This effect could be restored by the antioxidant vitamin C alone and in combination with vitamin E [31,32]. There were few other studies examining the post-prandial effects of Mg. However, chronic Mg supplementation for greater than 6 months was shown to have a beneficial effect on FMD in individuals >50 years or in overweight participants, although overall there was no effect of Mg found in this meta-analysis [24].

### 4.2. Serum Magnesium

There was a significant effect of Mg supplementation on serum Mg over time, in the present study, which was also observed in long-term supplementation studies [33]. Del Gobbo [5] found that circulating Mg (per 0.2 mmol/L increment) was associated with a 30% lower risk of CVD. In our study and in others in healthy individuals [23,34] or patients on haemodialysis [35], participants had baseline serum Mg concentrations within normal ranges. There is a need for further studies in participants with low serum Mg concentrations, to assess the effects on endothelial function, as measured by FMD. 

### 4.3. Strengths and Limitations

The strengths of this study include that the same operator undertook all FMD measurements for the study, who was blinded to the treatments at the time of measurement. Limitations of the study incude the small sample size and the lack of a placebo. As these were healthy volunteers with a normal FMD, we can only conclude that magnesium had no acute effect in this group and it might have a beneficial effect in people with CV disease or risk factors and a low FMD. This is the first study we are aware of that examined the effect of magnesium acutely. We previously showed that both sodium and potassium can have acute effects, so it was possible that magnesium might also have an acute as well as a chronic effect. Although we did not find an effect, we believe the area should be investigated further and that magnesium might be a useful supplement for people with impaired FMD.

## 5. Conclusions

The difference in FMD in the control group and the lack of change in the supplement group at 60-min post-drink suggests that Mg attenuates the reduction in FMD post-prandially. Further research is warranted to investigate the potential beneficial effect of MG supplementation on post-prandial endothelial function.

## Figures and Tables

**Table 1:** Baseline characteristics (*n* = 21).

	Range	Mean/Median	SD/IQR
Age (years)	19–75	39	16
Weight (kg)	45.8–100.9	59.2 (median)	14.2 (IQR)
Height (cm)	157–180	166.6	6.5
BMI (kg/m^2^)	17.9–31.9	21.1 (median)	5.6 (IQR)
SBP (mmHg)	92–134	113.5	13.8
DBP (mmHg)	65–90	76.2	7.2
MAP (mmHg)	75–101.7	88.5	8.7
24 h urine Mg excretion (mmol/L)	2.03–5.23	3.39	0.86
24 h urine Cr excretion (mmol/L)	3.64–20.0	8.82 (median)	3.19 (IQR)
Timepoint 0 brachial artery diameter (mm)	3.01–5.20	3.8	0.56
Baseline serum mg (mmol)	0.75–0.94	0.85	0.047

**Table 2:** Usual dietary intake as determined by 3-day weighed food records.

Nutrient	Range	Mean	SD
Mg Intake (*n* = 19) mg/day	138–510	339	106.7
Male Mg Intake (*n* = 4)	199–429	328	51.0
Female Mg Intake (*n* = 15)	138–510	343	97.7
Saturated Fat Intake (*n* = 19) g/day	6.7–57.9	27.7	13.7
Male Saturated Fat Intake (*n* = 4)	15.3–57.9	33.5	17.8
Female Saturated Fat Intake (*n* = 15)	6.7–46.4	26.1	12.7
PUFA Intake (*n* = 19) g/day	1.5–24.9	12.5	6.2
Male PUFA Intake (*n* = 4)	5.8–17.7	10.1	5.2
Female PUFA Intake (*n* = 15)	1.5–24.9	13.2	6.4

SD = standard deviation; Mg = Magnesium; and PUFA = polyunsaturated fat.

## Data Availability

The data presented in this study are available on request from the corresponding author. The data are not publicly available for ethical reasons.

## References

[1] Buttar H.S., Li T., Ravi N. (2005). Prevention of cardiovascular diseases: Role of exercise, dietary interventions, obesity and smoking cessation. Exp. Clin. Cardiol..

[2] Qu X., Jin F., Hao Y., Li H., Tang T., Wang H., Yan W., Dai K. (2013). Magnesium and the risk of cardiovascular events: A meta-analysis of prospective cohort studies. PLoS ONE.

[3] Chiuve S.E., Korngold E.C., Januzzi J.L., Gantzer M.L., Albert C.M. (2011). Plasma and dietary magnesium and risk of sudden cardiac death in women. Am. J. Clin. Nutr..

[4] Chiuve S.E., Sun Q., Curhan G.C., Taylor E.N., Spiegelman D., Willett W.C., Manson J.E., Rexrode K.M., Albert C.M. (2013). Dietary and plasma magnesium and risk of coronary heart disease among women. J. Am. Heart Assoc..

[5] Del Gobbo L.C., Imamura F., Wu J.H., de Oliveira Otto M.C., Chiuve S.E., Mozaffarian D. (2013). Circulating and dietary magnesium and risk of cardiovascular disease: A systematic review and meta-analysis of prospective studies. Am. J. Clin. Nutr..

[6] Rooney M.R., Alonso A., Folsom A.R., Michos E.D., Rebholz C.M., Misialek J.R., Chen L.Y., Dudley S., Lutsey P.L. (2020). Serum magnesium and the incidence of coronary artery disease over a median 27 years of follow-up in the atherosclerosis risk in communities (aric) study and a meta-analysis. Am. J. Clin. Nutr..

[7] Bagheri A., Naghshi S., Sadeghi O., Larijani B., Esmaillzadeh A. (2021). Total, dietary, and supplemental magnesium intakes and risk of all-cause, cardiovascular, and cancer mortality: A systematic review and dose-response meta-analysis of prospective cohort studies. Adv. Nutr..

[8] Yusuf S., Hawken S., Ounpuu S., Dans T., Avezum A., Lanas F., McQueen M., Budaj A., Pais P., Varigos J. (2004). Effect of potentially modifiable risk factors associated with myocardial infarction in 52 countries (the interheart study): Case-control study. Lancet.

[9] Dickinson K.M., Clifton P.M., Burrell L.M., Barrett P.H.R., Keogh J.B. (2014). Postprandial effects of a high salt meal on serum sodium, arterial stiffness, markers of nitric oxide production and markers of endothelial function. Atherosclerosis.

[10] Dickinson K.M., Clifton P.M., Keogh J.B. (2011). Endothelial function is impaired after a high-salt meal in healthy subjects. Am. J. Clin. Nutr..

[11] Dickinson K.M., Clifton P.M., Keogh J.B. (2014). A reduction of 3 g/day from a usual 9 g/day salt diet improves endothelial function and decreases endothelin-1 in a randomised cross_over study in normotensive overweight and obese subjects. Atherosclerosis.

[12] Dickinson K.M., Keogh J.B., Clifton P.M. (2009). Effects of a low-salt diet on flow-mediated dilatation in humans. Am. J. Clin. Nutr..

[13] Blanch N., Clifton P.M., Keogh J.B. (2015). A systematic review of vascular and endothelial function: Effects of fruit, vegetable and potassium intake. Nutr. Metab. Cardiovasc. Dis..

[14] Blanch N., Clifton P.M., Petersen K.S., Keogh J.B. (2015). Effect of sodium and potassium supplementation on vascular and endothelial function: A randomized controlled trial. Am. J. Clin. Nutr..

[15] Blanch N., Clifton P.M., Petersen K.S., Willoughby S.R., Keogh J.B. (2014). Effect of high potassium diet on endothelial function. Nutr. Metab. Cardiovasc. Dis..

[16] Australian National Health and Medical Research Council (2019). Heart, Stroke & Vascular Diseases.

[17] Versari D., Daghini E., Virdis A., Ghiadoni L., Taddei S. (2009). Endothelial dysfunction as a target for prevention of cardiovascular disease. Diabetes Care.

[18] Ellins E.A., Halcox J.P. (2011). Where are we heading with noninvasive clinical vascular physiology? Why and how should we assess endothelial function?. Cardiol. Res. Pract..

[19] Celermajer D.S., Sorensen K.E., Gooch V.M., Spiegelhalter D.J., Miller O.I., Sullivan I.D., Lloyd J.K., Deanfield J.E. (1992). Non-invasive detection of endothelial dysfunction in children and adults at risk of atherosclerosis. Lancet.

[20] Chacko S.A., Song Y., Nathan L., Tinker L., de Boer I.H., Tylavsky F., Wallace R., Liu S. (2010). Relations of dietary magnesium intake to biomarkers of inflammation and endothelial dysfunction in an ethnically diverse cohort of postmenopausal women. Diabetes Care.

[21] Song Y., Li T.Y., van Dam R.M., Manson J.E., Hu F.B. (2007). Magnesium intake and plasma concentrations of markers of systemic inflammation and endothelial dysfunction in women. Am. J. Clin. Nutr..

[22] Joosten M.M., Gansevoort R.T., Mukamal K.J., van der Harst P., Geleijnse J.M., Feskens E.J., Navis G., Bakker S.J., Group P.S. (2013). Urinary and plasma magnesium and risk of ischemic heart disease. Am. J. Clin. Nutr..

[23] Joris P.J., Plat J., Bakker S.J.L., Mensink R.P. (2017). Effects of long-term magnesium supplementation on endothelial function and cardiometabolic risk markers: A randomized controlled trial in overweight/obese adults. Sci. Rep..

[24] Marques B., Klein M., da Cunha M.R., de Souza Mattos S., de Paula Nogueira L., de Paula T., Correa F.M., Oigman W., Neves M.F. (2020). Effects of oral magnesium supplementation on vascular function: A systematic review and meta-analysis of randomized controlled trials. High Blood Press Cardiovasc. Prev..

[25] Liu M., Dudley S.C. (2020). Magnesium, oxidative stress, inflammation, and cardiovascular disease. Antioxidants.

[26] NHMRC (2006). Nutrient Reference Values for Australia and New Zealand: Including Recommended Dietary Intakes.

[27] Rosanoff A. (2021). Perspective: Us adult magnesium requirements need updating: Impacts of rising body weights and data-derived variance. Adv. Nutr..

[28] Sakai T., Sato B., Hara K., Hara Y., Naritomi Y., Koyanagi S., Hara H., Nagao T., Ishibashi T. (2014). Consumption of water containing over 3.5 mg of dissolved hydrogen could improve vascular endothelial function. Vasc. Health Risk Manag..

[29] Akbari C.M., Saouaf R., Barnhill D.F., Newman P.A., LoGerfo F.W., Veves A. (1998). Endothelium-dependent vasodilatation is impaired in both microcirculation and macrocirculation during acute hyperglycemia. J. Vascular. Surg..

[30] Kawano H., Motoyama T., Hirashima O., Hirai N., Miyao Y., Sakamoto T., Kugiyama K., Ogawa H., Yasue H. (1999). Hyperglycemia rapidly suppresses flow-mediated endothelium- dependent vasodilation of brachial artery. J. Am. College Cardiol..

[31] Title L.M., Cummings P.M., Giddens K., Nassar B.A. (2000). Oral glucose loading acutely attenuates endothelium-dependent vasodilation in healthy adults without diabetes: An effect prevented by vitamins c and e. J. Am. College Cardiol..

[32] Beckman J.A., Goldfine A.B., Gordon M.B., Creager M.A. (2001). Ascorbate restores endothelium-dependent vasodilation impaired by acute hyperglycemia in humans. Circulation.

[33] Hatzistavri L.S., Sarafidis P.A., Georgianos P.I., Tziolas I.M., Aroditis C.P., Zebekakis P.E., Pikilidou M.I., Lasaridis A.N. (2009). Oral magnesium supplementation reduces ambulatory blood pressure in patients with mild hypertension. Am. J. Hypertensi..

[34] Cosaro E., Bonafini S., Montagnana M., Danese E., Trettene M.S., Minuz P., Delva P., Fava C. (2014). Effects of magnesium supplements on blood pressure, endothelial function and metabolic parameters in healthy young men with a family history of metabolic syndrome. Nutr. Metab. Cardiovasc. Dis..

[35] Mortazavi M., Moeinzadeh F., Saadatnia M., Shahidi S., McGee J.C., Minagar A. (2013). Effect of magnesium supplementation on carotid intima-media thickness and flow-mediated dilatation among hemodialysis patients: A double-blind, randomized, placebo-controlled trial. Eur. Neurol..

